# Phytosphingosine exhibits an anti-epithelial–mesenchymal transition function by the inhibition of EGFR signaling in human breast cancer cells

**DOI:** 10.18632/oncotarget.20783

**Published:** 2017-09-08

**Authors:** Hye-Min Kang, Han-Sun Son, Yan-Hong Cui, BuHyun Youn, Beomseok Son, Nagendra Kumar Kaushik, Nizam Uddin, Jae-Seong Lee, Jie-Young Song, Neha Kaushik, Su-Jae Lee

**Affiliations:** ^1^ Department of Biological Science, College of Science, Sungkyunkwan University, Suwon, Republic of Korea; ^2^ Department of Life Science, College of Natural Sciences, Hanyang University, Seoul, Republic of Korea; ^3^ Department of Integrated Biological Science, Pusan National University, Busan, Republic of Korea; ^4^ Plasma Bioscience Research Center, Department of Electrical and Biological Physics, Kwangwoon University, Seoul, Republic of Korea; ^5^ Division of Radiation Cancer Research, Korea Institute of Radiological & Medical Sciences, Seoul, Republic of Korea

**Keywords:** phytosphingosine, epithelial-mesenchymal transition, cancer stem cells, epidermal growth factor receptor

## Abstract

The lack of effective anti-metastatic drugs for the eradication of breast cancer stem cells within tumors, which are often resistant to chemotherapy and radiotherapy, creates a major obstacle during metastatic breast cancer therapy. Although D-ribo-phytosphingosine (PHS) is well known to activate protein kinase (MAPK)-mediated apoptosis, its possible role towards the metastasis signaling mechanisms underlying the epithelial-mesenchymal transition (EMT) remains largely unknown. In this report, we investigate the anti-metastatic potential of the natural sphingolipid PHS for the targeting of breast cancer cells as well as breast stem-like cells *in vitro*. We showed that PHS led to suppression of migratory potential, spheroid formation, CD44^high^/CD24^low^ subpopulation as well as stem cell- and EMT-associated protein expression in basal type highly malignant breast cancer cell lines. In addition, PHS-based inhibition of EMT was attributable to the downregulation of the EGFR/JAK1/STAT3 signaling axis, as validated by immunoprecipitation assays and breast tumorigenesis mice models. This study demonstrate that PHS can target metastatic tumors with dual specificity (EMT and cancer stem-like cells) and therefore may be serve as a promising candidate for breast cancer treatments.

## INTRODUCTION

It is firmly established that the overexpression and activation of human epidermal growth factor receptor (EGFR), as reported in up to 30% of solid tumors (including breast, colorectal, lung, gastric, head and neck cancer, and glioblastomas), generally correlate with a poor prognosis [1]. Human EGFR is overexpressed in nearly all subtypes of breast cancer; however, it is more frequently overexpressed in triple-negative or basal-type breast cancers, which are highly aggressive and resistant to existing therapies [2–4]. The main reason behind this is the resistance of breast cancer stem cells towards conventional chemotherapy or radiation therapy, as they can't eliminate most bulk breast cancer cells and are responsible for further relapse of breast cancer. Thus, EGFR has emerged as a therapeutic target in cases of aggressive breast tumors, for which there are no effective targeted therapies available so far. In many types of cancer, EGFR alterations occur at an advanced stage of malignancy, characterized by metastatic competence [5]. Several lines of evidence suggest that EGFR and its downstream signaling pathway regulate the epithelial-mesenchymal transition (EMT) and tumor invasion process [6, 7]. EMT is depicted by the loss of cell–cell junctions, cytoskeletal rearrangements, increased cell motility [8, 9]. Particularly, certain epithelial cells that undergo an EMT acquire stem-like properties [10]. In assistance with these properties, several EMT biomarkers have been recognized, some of which have been practiced to detect EMT in clinical samples. Hence, an EGFR-targeted therapy may be a promising approach for overcoming resistance to chemotherapy and radiotherapy in breast cancers.

It has been proposed that sphingolipid metabolites such as ceramides, sphingosines, and sphingosine 1-phosphates are crucial mediators of apoptotic cell death [11]. The activation of the JNK pathway is involved in ceramide-induced apoptosis [12]. Ceramide can be further metabolized by ceramidase to sphingosine, which can then be phosphorylated by sphingosine kinase to form sphingosine 1-phosphate [13]. Whereas sphingosine induces apoptosis in various cell types (30), sphingosine 1-phosphate antagonizes the apoptotic actions of ceramide [14]. Given the well-established roles of these three sphingolipid metabolites in apoptosis, other sphingolipid derivatives may also contribute to controlling apoptosis. Among these a derivative, D-ribophytosphingosine (PHS) is one of the most extensively distributed natural sphingolipid; it is abundant in fungi and plants and is also found in animals, including humans. The structure of PHS is very similar to the sphingosine; PHS possesses a hydroxyl group at C-4 of the sphingoid long-chain base, whereas sphingosine has a trans-double bond between C-4 and C-5. Recent report claims that PHS exerts strong cytotoxic effects on chinese hamster ovary cells and modulates the muscarinic acetylcholine receptor-mediated signal transduction pathway [15]. The functional mechanism of the anticancer activity of PHS has been widely studied [16, 17]. However, the roles of PHS regarding its anti-metastatic capabilities remain relatively unexplored.

In the present study, we investigate for the first time the possible role of PHS towards anti-EMT phenomenon and the underlying mechanisms in basal-type breast cancers. Specifically, we show that the deactivation of EGFR pathway is critical during PHS-decreased EMT. The successful elimination of the breast-like cancer stem cell (CSC) populations within tumors is a major challenge in breast cancer research. Given that PHS appears to target dual-specificity (EMT and CSC) to prevent breast cancer metastasis, understanding the signaling mechanisms of PHS-attenuated metastasis may provide a better understanding of preventing disease in human systems.

## RESULTS AND DISCUSSION

### PHS inhibits EMT in breast cancer cells

**Figure 1:**
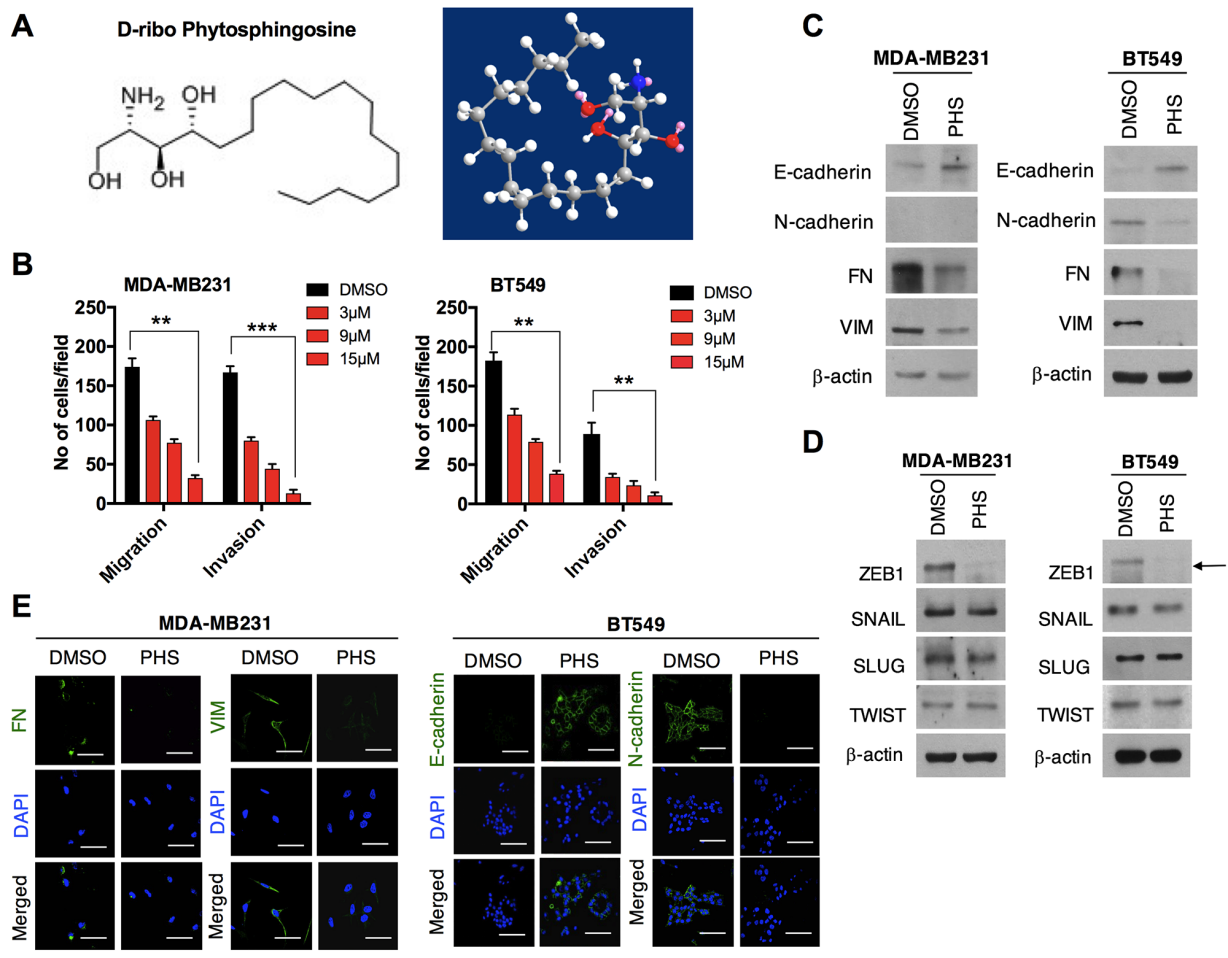
PHS decreases EMT in breast cancer cells **(A)** Chemical and molecular structure of phytosphingosine (PHS) generated by ChemDraw professional software. **(B)** Invasion and migration assays of PHS-treated MDA-MB231 and BT549 basal-type breast cancer cells at various concentrations. **(C)** Western blot analysis of the EMT markers Fibronectin (FN), Vimentin (VIM), E-cadherin and N-cadherin in MDA-MB231 or BT549 cells after a DMSO or PHS treatment (10μM). **(D)** Western blot analysis of EMT transcription factors of SNAIL, SLUG, ZEB1 and TWIST in PHS (10μM)-treated MDA-MB231 and BT549 cells. **(E)** Immunofluorescence staining of EMT surface markers in PHS-treated MDA-MB231 and BT549 cells at a concentration of 10μM. DAPI, 4,6-diamidino-2-phenylindole. DMSO is used as a vehicle control here at a concentration similar to that of PHS. β-actin was used as a loading control. Scale bar = (100μm). Error bars represent the mean ± S.D. of triplicate samples. *p < 0.05, **p < 0.01 and ***p < 0.001.

Here, we used a natural sphingolipid PHS to treat breast cancer cells, as shown in Figure 1A. It has been recognized that during tumorigenesis, EMT may increase the motility and invasiveness of cancer cells, with subsequent malignant transformation promoting EMT [18]. Thus, we aimed to determine the effects of PHS on the process of EMT in breast cancer cells. To this end, we initially conducted Boyden chamber invasion and migration assays in two of the most aggressive basal-type breast cancer MDA-MB231and BT549 cells with different concentrations of PHS. Prior to these experiments, we conducted a viability test using MDA-MB231 and BT549 cells. The viability test and PI analysis showed that PHS did not affect cell metabolic viability levels in a dose and incubation time-dependent manner (Supplementary Figures 1A & 1B). Transwell chamber assays showed significant decreases in the migration and invasion of MDA-MB231 and BT549 cells in a dose-dependent manner (Figure 1B & Supplementary Figure 1C). We also noted that PHS induced the significant dose-dependent inhibition of migration in both cell types, starting with concentrations of 3μM-15μM at 48 h post-treatment in wound-healing assays (Supplementary Figure 1D). As EMT is evidenced through a loss of epithelial markers and a gain of mesenchymal markers, we analyzed EMT markers and EMT-inducing transcriptional activators. Sustained E-cad induction and decreases in VIM and FN in both cell lines indicate that PHS can effectively inhibit the EMT process in breast cancer cells (Figure 1C). In addition, among all EMT-transcriptional activators tested, ZEB1 showed a greater decrease after PHS exposure in both cell lines at concentrations of 10μM (Figure 1D). Interestingly, si-RNA mediated targeting of the ZEB1-decreased induction of EMT, as observed in BT549 cells (Supplementary Figures 1E & 1F). Moreover, the immunofluorescence data revealed that FN and VIM were decreased in MDA-MB231 cells after the PHS treatment, whereas for the BT549 cells, E-cad was increased with a reduction in the N-cad levels (Figure 1E). Therefore, these results indicate that PHS inhibits the migratory capability of basal-type breast cancer cells at sub-toxic concentrations.

### PHS suppresses the self-renewal ability in breast cancer cells

**Figure 2:**
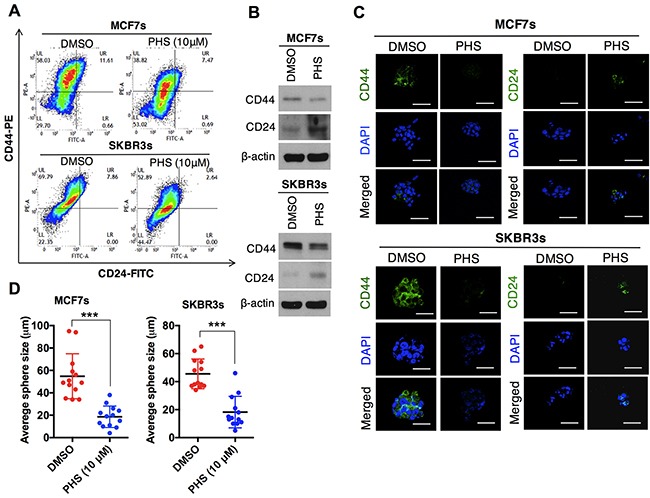

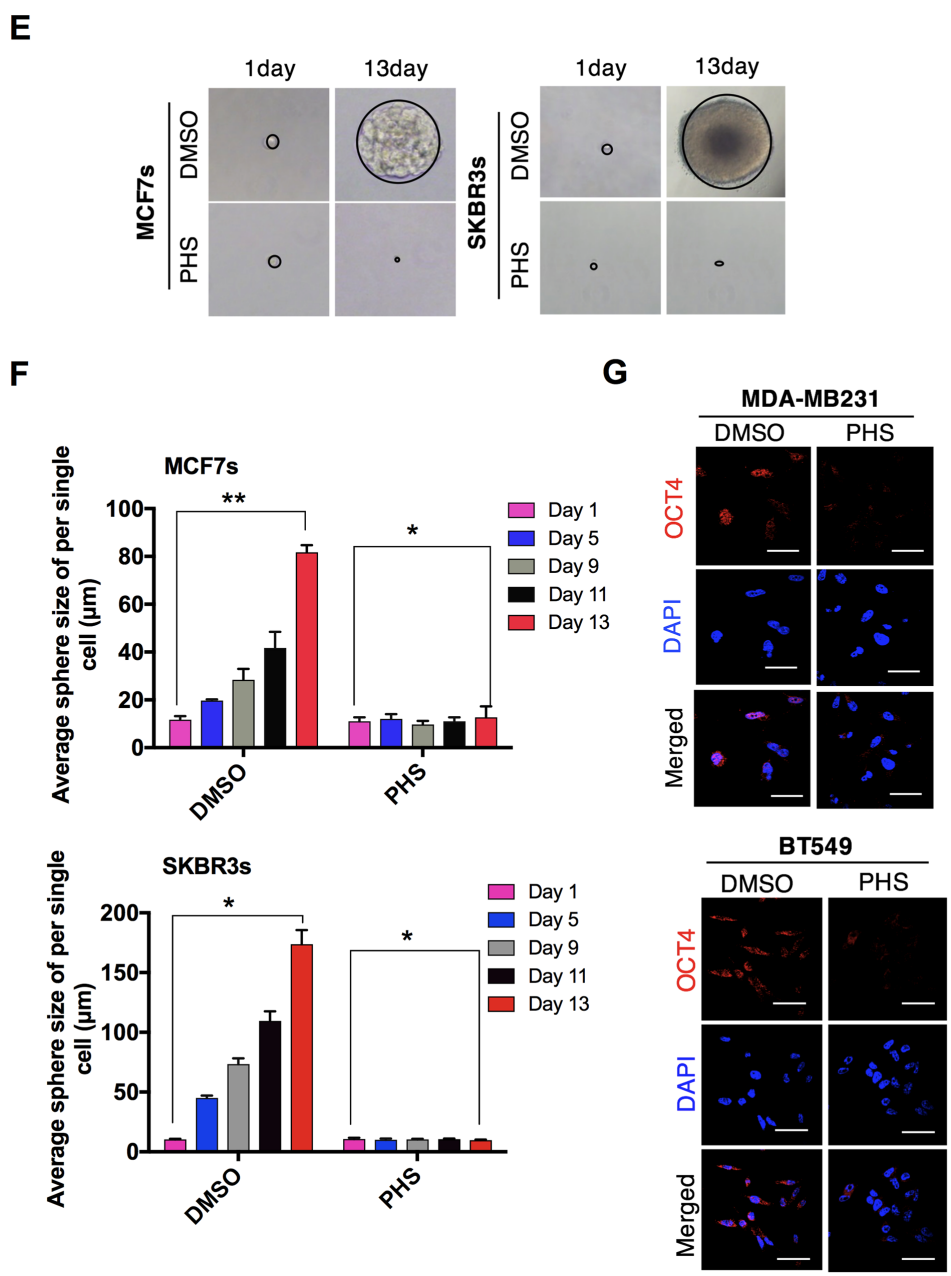
PHS suppresses the self-renewal ability of breast-like stem cell-populations **(A)** FACS analysis for the CD44-PE and CD24-FITC expression levels in DMSO or PHS (10μM)-treated MCF7 (upper panel) and SKBR3 (lower panel) mammospheres. **(B)** Western blot analysis results of CD44 and CD24 protein levels in DMSO or PHS (10μM)-treated MCF7 and SKBR3 mammospheres. β-actin was used as a loading control. **(C)** Immunofluorescence staining of CD44 and CD24 expression levels in DMSO or PHS (10μM)-treated MCF7and SKBR3 mammospheres. **(D)** Quantification of the sphere-forming capabilities of MCF7 and SKBR3 sphere cells after a treatment with PHS (10μM) or a control vehicle, DMSO. **(E)** Clonal assay results of MCF7 and SKBR3 sphere cells 13 days after a treatment with PHS (10μM) or the control vehicle DMSO. **(F)** Changes in the sphere size of single cell in the presence of DMSO and PHS were monitored at different time points in both cells and are plotted on a graph. **(G)** Immunofluorescence staining of the OCT4 expression levels in DMSO or PHS (10μM)-treated MCF7 and SKBR3 spheres. Scale bar = (100μm). Error bars represent the mean ± S.D. of triplicate samples. *p < 0.05, **p < 0.01 and ***p < 0.001.

In cases of breast cancer, CD44^high^/CD24^low^ cell surface phenotypes are enriched in stem-like breast cancer cells. These stem cells contribute to the acquisition of chemotherapy resistance across a wide range of malignancies [19–21]. To this end, the surface markers (CD44^high^/CD24^low^) in the MCF7 and SKBR3 mammospheres were subjected to flow cytometry analyses. The results showed that the CD44^high^/CD24^low^ population was significantly attenuated in both MCF7 and SKBR3 sphere cell lines after the PHS (10μM) treatment (Figure 2A). The dose-dependent treatment of PHS significantly decreased the CD44 positive population in both cell types, as shown in Supplementary Figure 2A. In addition, a western blot analysis showed that the CD44 protein levels were downregulated with an increase in the CD24 levels in the MCF7 and SKBR3 mammospheres after the PHS (10μM) treatment (Figure 2B). Consistent with these findings, immunofluorescence staining confirmed that CD44 was decreased in these cells, whereas the expression of CD24 was increased after PHS exposure at a concentration of 10μM (Figure 2C). Earlier findings suggested that a subpopulation (CD44^high^/CD24^low^) of breast cancer cells had stem-like cell properties, such as self-renewal or sphere-forming capabilities [19, 20, 22]. To investigate whether this CD44^high^/CD24^low^ subpopulation of cancer cells also shares stem-like cell proliferation capabilities, we performed self-renewal and sphere-forming assays with MCF7 and SKBR3 mammospheres. Of note, PHS effectively decreased the sizes of the spheres in both types of cells at concentration of 10μM (Figure 2D & Supplementary Figure 2B). Single-cell analysis results showed that MCF7 and SKBR3 attenuated the self-renewal capacities, as visualized in the days after a post-incubation PHS treatment (Figures 2E & 2F & Supplementary Figure 2C). Apart from CD44, the cancer stem cells exhibited high levels of the expression of SOX2, OCT4, β-catenin and NOTCH2 [23–25]. When the expression levels of these proteins were analyzed in MCF7 and SKBR3 mammospheres by western blot analyses, it was found that PHS decreased the expression of OCT4 most remarkably among all stem cell maintenance regulators tested in both sphere-cultured cells to a greater extent with a similar dose treatment (Supplementary Figure 3A). In agreement with these results, immunofluorescence staining confirmed that OCT4 expression levels were noticeably decreased after the PHS treatment in mammospheres (Figure 2G). To validate the role of OCT4 more strongly in this phenomenon, we undertook the silencing of OCT4 in MCF7s and SKBR3s and performed single-cell assay. OCT4 silencing noticeably decreased the CD44 levels with increased CD24 levels in both sphere cell lines (Supplementary Figure 2D). Moreover, the self-renewal capacity was reduced in MCF7s and SKBR3s after OCT4 silencing (Supplementary Figure 2E). Similar changes were observed in MDA-MB231 and BT549 cells with OCT4 silencing (Supplementary Figure 2F). Taken together, these results indicate that PHS reduces the self-renewal capability as well as the expression levels of stemness regulators in breast cancer cells.

### PHS confers sensitivity to CSC-like populations in basal-type breast cancer cells

**Figure 3:**
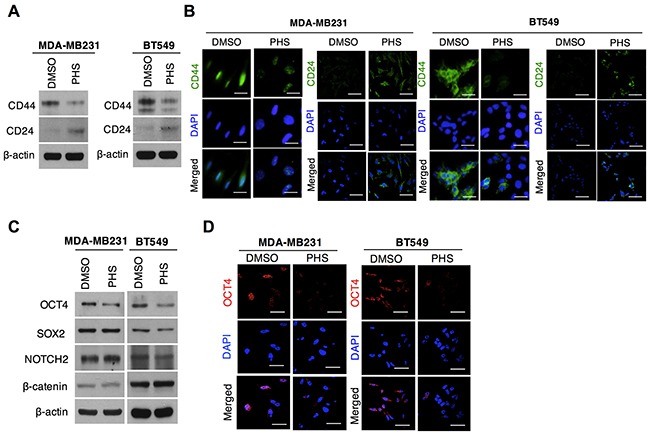
PHS blocks stem cell populations in basal-type breast cancer **(A)** Western blot analysis results of CD44 and CD24 protein levels in MDA-MB231 and BT549 breast cancer cells treated with or without PHS (10μM). **(B)** Immunofluorescence staining of CD44 and CD24 expression levels in DMSO or PHS (10μM)-treated MDA-MB231 and BT549 cells. **(C)** Western blot analysis results for SOX2, OCT4, NOTCH2 and β-catenin in MDA-MB231 and BT549 cells treated with or without PHS (10μM). **(D)** Immunofluorescence staining of OCT4 expression levels in DMSO or PHS (10μM)-treated MDA-MB231 and BT549 cells. Scale bar = (100μm).

Most breast cancer stem cells (CSCs) enriched with the CD44^high^/CD24^low^ phenotype can also be found in basal-like breast tumors [26]. Moreover, this phenotype has been observed in ‘triple-negative’ breast cancer cells, which are highly resistant to chemotherapy [27]. Given these findings, we therefore asked whether the stem-like cell populations that survive in basal-type breast cancer cells continue to maintain stemness after a PHS treatment. To locate these cells, we checked CD44 population in two basal-type breast cancer cells: MDA-MB231 and BT549 cells. A flow cytometry analysis showed that the CD44 positive population decreased with an increase in the PHS exposure dose (Supplementary Figure 3B). Both cells showed a great decrease in CD44 levels with an increase in CD24 levels, as measured by western blot analyses after the PHS (10μM) treatment (Figure 3A). Consistent with this, an immunocytochemical analysis showed that in both MDA-MB231 and BT549 cells, PHS strongly suppressed CD44 expression levels (Figure 3B). Reduced expression levels of OCT4 protein, a CSC maintenance regulator, were also noted in both cell types (Figures 3C & 3D), in agreement with the above findings. Collectively, we observed an identical association between mammospheres and basal-type breast cancer cell lines with regard to a PHS treatment, with a significant decrease in the CD44 phenotype.

### PHS blocks the metastatic ability of breast cancer cells *in vivo*

**Figure 4:**
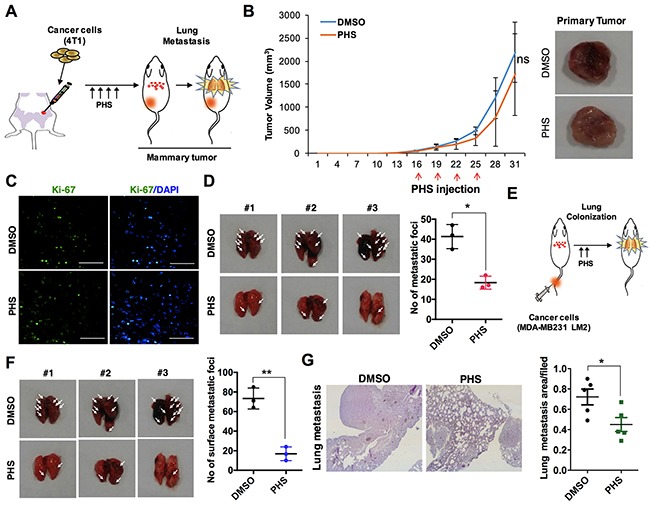
PHS suppresses the tumorigenicity of breast cancer cells *in vivo* **(A)** Schematic of the experimental procedure of the mammary fat pad injection of 4T1 mouse breast cancer cells and treatment with a vehicle DMSO or PHS. **(B)** Typical images of primary tumors (right panel) and tumor growth curves (left panel). Green fluorescent protein (GFP)-labeled 4T1 cells were injected into the mammary fat pads of nude mice (n=5) and were then treated four times with PHS or a vehicle by i.p. injections. The graph represents the tumor volume of DMSO- and PHS-treated mice. **(C)** Ki-67 positive immunostaining in PHS- or vehicle DMSO-treated tumor tissue sections. Scale bar = (50μm). **(D)** Representative images and quantification of lung metastatic foci generated by GFP-labeled metastatic 4T1 cells after mammary fat pad injections. **(E)** Schematic experimental procedure of the tail vein injection of cancer cells into athymic nude mice and treatment with a vehicle or PHS. **(F)** Quantification of lung metastatic foci generated by GFP-labeled metastatic MDA-MB231 LM2 cells after tail vein injections (n=5). **(G)** H&E staining (left panel) of lung tissue sections after mammary tail vein injections in DMSO- and PHS-treated mice. Typical graphical representation of the lung metastatic area per field in treated and control tissues. Error bars represent mean ± S.D. of triplicate samples. *p < 0.05, and **p < 0.01.

Intrigued by the persistence of the decreased mesenchymal markers associated with EMT, we subsequently questioned whether PHS could overcome the metastasis of breast cancer *in vivo*. To this end, GFP-labeled metastatic 4T1 breast cancer cells were transplanted into the mammary fat pads of athymic balb/c nude mice (n=5) and PHS was used as a treatment four times on two alternating days, as indicated in Figure 4A. In terms of tumor volumes, we found that a treatment with PHS did not significantly attenuate primary tumor formation in mammary fat pads (Figure 4B). Indeed, compared to a control vehicle (DMSO), PHS-treated mice showed no decrease in the number of proliferating cells, as detected by Ki67 positive cell staining (Figure 4C). However, lung metastasis was markedly decreased in PHS-treated tumors as compared to vehicle-treated cases (Figure 4D). Having found that PHS has strong potential to suppress lung metastasis rather than to retard tumor growth (possibly due to the inhibition of primary tumor formation) through fat pad injection trials, in an effort to clarify this further, we inoculated metastatic GFP-labeled MDA-MB231 LM2 cells directly into athymic nude mice (n=5) by means of intravenous injections (Figure 4E). The mice were then treated with a vehicle or PHS by i.p. injections four times on alternating days. As expected, the numbers of metastatic foci were effectively decreased in the PHS-treated mice compared to their vehicle-treated counterparts (Figure 4F). Histochemical staining of lung tissue sections confirmed that the degree of the formation of lung metastatic lesions was decreased after the PHS treatment in mice (Figure 4G). Collectively, these data suggest that PHS mitigates the metastatic capability of breast cancer cells *in vivo*.

### PHS may bind to EGFR and suppress EMT through its deactivation

**Figure 5:**
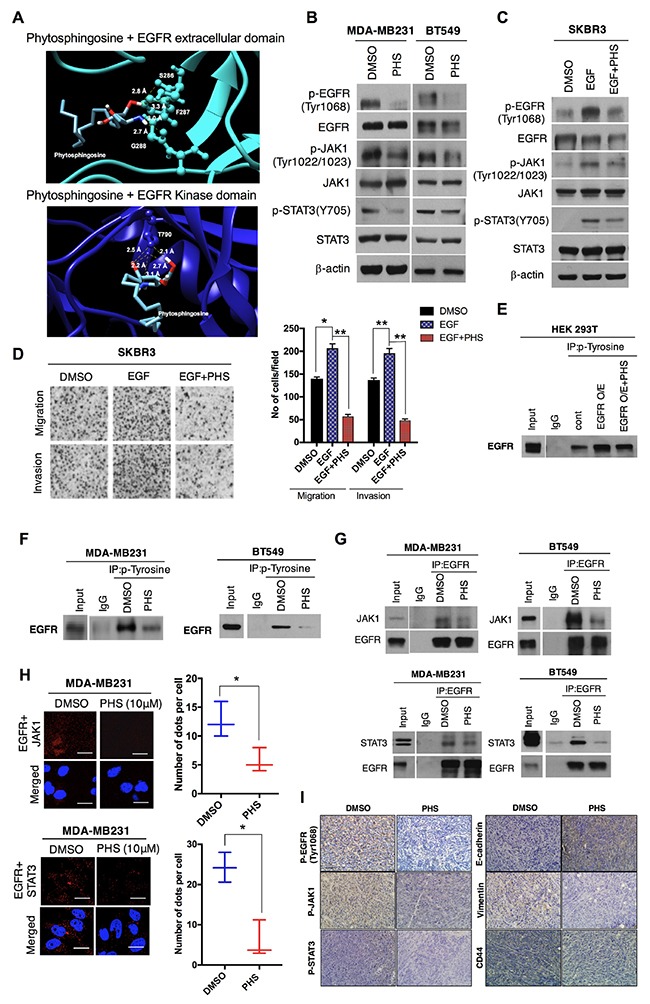
PHS may bind to EGFR and suppress EMT **(A)** Molecular docking of PHS with the extracellular and kinase EGFR domain. **(B)** Western blot analysis results related to p-EGFR (Tyr1068), EGFR, JAK, p-JAK1, STAT3, and p-STAT3 (Y705) levels in MDA-MB231 and BT549 breast cancer cells treated with or without PHS (10μM). **(C)** Western blot analysis results of pEGFR (Tyr1068), EGFR, p-JAK1, JAK1, STAT3, and p-STAT3 (Y705) levels in EGF (100ng/ml) stimulated SKBR3 breast cancer cells treated with or without PHS (10μM). β-actin was used as a loading control. **(D)** Images of migration and invasion assays in Transwell plates after EGF and EGF plus a PHS treatment (10μM) in SKBR3 cells. **(E)** Anti-phospho tyrosine immunoprecipitates from PHS (10μM)-treated EGFR-overexpressing HEK293T cells were immunoblotted for the EGFR antibody. **(F)** Anti-phospho tyrosine immunoprecipitates from PHS-treated MDA-MB231 and BT549 cells were immunoblotted for EGFR. **(G)** Anti-EGFR immunoprecipitates from PHS-treated MDA-MB231 and BT549 cells were immunoblotted for JAK1 and STAT3. **(H)** MDA-MB231 cells were fixed and incubated with mouse anti-EGFR together with rabbit anti-JAK1 or rabbit anti-STAT3, followed by an in-situ PLA analysis. Representative confocal images and a graph of cells with PLA-positive signals are shown; the numbers of dots per cell were counted using ImageJ software. Scale bar = (100μm). **(I)** IHC staining of p-EGFR (Tyr1068), p-JAK1, p-STAT3, E-cadherin, Vimentin and CD44 expression levels in PHS- or control-vehicle-treated primary tumors formed through mammary fat pad injections of 4T1 breast cancer cells. Scale bar = (100μm).

Given the role of EGFR activation in the progression of breast cancer, we subsequently undertook molecular docking to predict the PHS binding parameters with human EGFR. Direct evidence from the molecular modeling of the human EGFR extracellular domain (4UV7) or the kinase domain (4ZAU) suggested that PHS could bind to EGFR and that it shows affinity for the extracellular and kinase domain of EGFR at -4.5 kcal·mol^-1^ and -4.7 kcal·mol^-1^, respectively. PHS was capable of hydrogen bonding with Ser286, Phe287, and Gly288 of the EGFR extracellular domain, whereas it formed hydrogen bonds only with Thr790 in the EGFR kinase domain (Figure 5A). Simultaneously, a crosslinking assay revealed that PHS did not trigger EGFR homo-dimerization (Supplementary Figure 4A), suggesting that PHS blocks EGFR-induced tumor progression by inhibiting phosphorylation rather than dimerization. Next, we subsequently examined whether a PHS treatment could alter the activity of EGFR targets (EGFR-family kinases). When we treated MDA-MB231 and BT549 cells with PHS (10μM), we observed that the PHS treatment efficiently decreased the phosphorylation of EGFR-family kinases (EGFR, JAK1, and STAT3) in both cell lines (Figure 5B). Furthermore, AKT and MAPK phosphorylation were also decreased in both cell lines after a PHS (10μM) treatment, consistent with the decreased signaling through EGFR (Supplementary Figure 5A). Similar changes were observed when PHS was used to treat EGF (100ng/ml, a well-known EMT inducer)-stimulated SKBR3 breast cancer cells (Figure 5C and Supplementary Figure 5B). Boyden chamber assays revealed that the PHS treatment also decreased the migration and invasion of EGF-stimulated SKBR3 cells (Figure 5D). Therefore, we then sought to determine how PHS may suppress EGFR activity levels and inhibit the EGFR downstream signaling pathway. The phosphorylation of EGFR at certain sites blocks the activity of receptor tyrosine kinases, and the phosphorylation of tyrosine residue 1068 has been shown directly to interact with STAT3 and regulate its phosphorylation [28]. We initially confirmed the effect of PHS in EGFR-overexpressing HEK293T cells. Immunoprecipitation with an anti-phospho tyrosine antibody decreased EGFR phosphorylation in EGFR-overexpressing 293T cells after a PHS treatment (Figure 5E). To determine whether PHS could also regulate EGFR phosphorylation in basal-type breast cancer cells, we treated MDA-MB231 and BT549 cells with PHS at similar concentrations and found reduced EGFR phosphorylation levels at Tyr1068 in both cell lines (Figure 5F). In addition, immunoprecipitation with an EGFR antibody also decreased JAK1 and STAT3 levels in both cell lines after the PHS treatment (Figure 5G). To confirm our observations, we used a PLA technique which enables the visualization and quantification of specific protein-protein interaction events in situ with unique specificity [29, 30]. It is based on the use of two primary antibodies raised in different species that recognize the antigens of interest. Subsequently species-specific secondary antibodies, called PLA probes, each with a unique short DNA strand attached to it, are added. Notably, the PHS treatment effectively attenuated the interaction of EGFR with JAK1 and STAT3 in MDA-MB231 cells, as determined by the PLA technique (Figure 5H). Moreover, an immunohistochemical (IHC) analysis revealed decreased pEGFR levels and related downstream network pathway expression levels in PHS-treated tumors formed by a fat pad injection of 4T1 breast cancer cells (Figure 5I). We also observed altered EMT markers and CD44 expression in PHS-treated tumors compared to a vehicle (Figure 5I). To clarify this, we also assessed possible changes in the target genes of metastasis related to the EGFR/JAK/STAT pathway. As expected, PHS alters the expression levels of genes which are directly regulated by STAT3 (Supplementary Figure 5C). These findings indicate that PHS can significantly inhibit EGFR activity through direct binding, implying its role in the PHS-induced decrease of the EMT process in breast cancer cells.

## MATERIALS AND METHODS

### Chemical reagents and antibodies

PHS was purchased from Avanti Polar lipids (Alabaster, AL). Antibodies specific to p-STAT3 (Tyr705, ##4113), p-AKT (Ser473, #4060), p-AKT (Thr308, #9275), p-P38 MAPK (Thr180/Tyr182, #9211), p38 MAPK (#9212), p-SAPK/JNK (Thr183/Tyr185, #4668), p-EGFR (Tyr1068, #2234), JNK (#9252), and SNAIL (#3879) were purchased from Cell Signaling Technology (Beverly, MA, USA). Antibody specific to NOTCH2 (07-1234) was purchased from Millipore Corp (Billerica, MA, USA). Antibodies specific to SOX2 (sc-20088), OCT4 (sc-9081), AKT (sc-7126), STAT3 (sc-7179), p-ERK (sc-7383), p-JAK1 (Tyr1022/Tyr1023, sc-101716), EGFR (sc-03, sc-120), Fibronectin (sc-69681), SLUG (sc-10437), TWIST (sc-15393), E-cadherin (sc-8426), and p-Tyrosine (sc-508) were purchased from Santa Cruz Biotechnology (Santa Cruz, CA). Vimentin (ab8978), Ki67 (ab15580), CD44 (ab157107) and CD24 (ab31622) were obtained from Abcam whereas anti-ZEB1 (HPA027524) and β-actin (#A1978) antibody were purchased from Sigma-Aldrich. Antibodies specific to β-catenin (#610153) and N-cadherin (#610921) were purchased from BD Transduction Laboratories. EGF, bFGF, and accutase were obtained from Sigma (St. Louis, MO, USA).

### Cell culture and drug treatment

The human breast cancer cell lines MCF7, SKBR3, MDA-MB231 and BT549, and HEK293T embryonic kidney cells were purchased from the American Type Culture Collection (Manassas, VA). MCF7 and SKBR3 are luminal type whereas MDA-MB231 and BT549 cells are basal-type breast cancer cell lines. SKBR3, MDA-MB231 and BT549 were grown in RPMI-1640 medium while MCF7 cells were grown in MEM supplemented with 10% fetal bovine serum, penicillin, and streptomycin at 37°C in a humidified incubator with 5% CO_2_. HEK293T cells were cultured in DMEM containing 10% FBS. Initially, all cells were treated with plasmocin-mycoplasma-elimination reagent (Invivogen, USA) to retain these cells free from mycoplasma contamination. All breast cancer cells were frozen and stored in liquid nitrogen. These cell lines were maintained less than 5 months. For sphere culture conditioned medium, breast cancer cells were cultured in serum-free DMEM-F12 media (Invitrogen, Seoul, Korea) as described previously [31]. Stock solution of PHS was prepared in DMSO without further characterization and directly applied in the cell culture experiments.

### Small interfering RNA transfection

RNA interferences of ZEB1 and OCT4 were performed using 21-base siRNA duplexes purchased from Genolution Pharmaceuticals, Inc. The sense strand nucleotide sequences are as follows: ZEB1 siRNA (ACACAUAAGCAGUAAGAAAUU) and OCT4 siRNA (GCUUCAAGAACAUGUGUAAUU). A control siRNA specific to the green fluorescent protein (GFP) DNA sequence GUUCAGCGUGUCCGGCGAGUU was used as a negative control. For transfection, cells were seeded at 70% confluency and siRNA duplexes (200nM) were introduced into the cells using Lipofectamine 2000 (Invitrogen) according to the manufacturer's recommendations.

### Transduction

The diluted blank vectors and EGFR-expressing plasmids including plus regent, followed by incubation at room temperature for 15 minute (min) were independently mixed with Lipofectamine® reagent, followed by further incubation at room temperature for 15 min. Afterwards, cells were further incubated for 48 hours (hr) and processed as per required experiments. pCDNA6A-EGFR WT was a gift from Mien-Chie Hung (Addgene plasmid # 42665) [32].

### Cell viability studies

The cytotoxicity of the lipids was determined in breast cancer cells using MTT (Sigma-aldrich) colorimetric assays and sample absorbance was recorded at 570 nm. This experiment was performed in triplicate (n=3).

### Flow cytometric analysis

To assess cell death, cells were stained with propidium iodide (PI; Sigma, 50ng/mL) and analyzed using BD FACSVerse with the FACS suite software. To quantify the CD44 or CD24 population, cells were stained with PE-mouse anti-human CD44 or FITC mouse anti-human CD24 in phosphate buffered saline (PBS) containing 0.5% bovine serum albumin (BSA) and 2mM ethylenediaminetetraacetic acid (EDTA). As a control, cells were stained with FITC or the PE mouse IgG1κ isotype (BD, Seoul, Korea). Stained cells were then analyzed by FACS analysis through a BD FACSVerse (BD Biosciences).

### Matrigel invasion and migration assays

For the invasion assays, we used Transwell Boyden chambers with inserts that were precoated with 10 mg/ml growth factor-reduced Matrigel (BD Biosciences). 2 × 10^4^ cells per well were placed in the upper chamber of a Transwell chamber in serum-free DMEM. The cells were then allowed to migrate toward the lower chamber, in which DMEM containing 10% FBS was placed, for 20–24 hr at 37°C. Migrated cells were fixed and stained using a Diff-Quick Staining Kit (Fisher, Pittsburgh, USA) and were photographed using a Nikon light microscope [33]. For each sample, at least 5 randomly chosen independent field in different locations or cells were acquired. The extent of invasion was expressed as the average number of cells per field. In the migration assays, we used similar inserts without the Matrigel coating.

### Immunofluorescence

Briefly, after fixation with 4% paraformaldehyde, cells were permeabilized with 0.1% Triton X-100 in PBS. Following cell fixation, immunofluorescence staining for CD44, OCT4, fibronectin (FN), E-cadherin (E-cad), Vimentin (VIM) and N-cadherin (N-cad) was performed at an antibody dilution of 1:200 in blocking buffer containing 10% FBS and 1% NP-40 in PBS. These cells were stained with anti-rabbit or anti-mouse Alexa Fluor® 488 and counterstained with 4, 6-diamidino-2-phenylindole (DAPI; Sigma, USA). To visualize the CD24 stained cells, we used anti-CD24-FITC antibody. The stained images were observed under 60X magnification using a fluorescence confocal microscope (Nikon).

### Immunoprecipitation

Cells were lysed with cold cell lysis buffer [40 mM Tris–HCl (pH 8.0), 120mM NaCl, 0.1% Nonidet-P40], and the lysates were precleared with protein A-Sepharose (Sigma-Aldrich Co). Then the resulting supernatant fractions were incubated with a primary antibody at 4°C for 3 hr. Immune complexes were collected using protein A-Sepharose and washed 3 times with lysis buffer, and further dissolved in SDS sample buffer and detected using immunoblotting.

### Western blot analysis

Briefly, total cell lysates were prepared by extracting proteins with lysis buffer [40 mM Tris–HCl (pH 8.0), 120 mM NaCl, 0.1% Nonidet-P40] supplemented with protease inhibitors. Proteins were separated by SDS-PAGE and transferred to a nitrocellulose membrane (Amersham, Arlington Heights, IL). The membrane was blocked with 5% non-fat dry milk in tris-buffered saline, and incubated with primary antibodies for overnight at 4°C. The blots were developed using peroxidase-conjugated secondary antibody, and proteins were visualized by enhanced chemiluminescence (ECL) procedures (Amersham, Arlington Heights, IL) using the manufacturer's protocol.

### Sphere-forming and self-renewal assays

For sphere forming assays, size of PHS-treated spheres was monitored on day 1-4 using Motic Images Plus 2.0 software in three randomly chosen fields. We measured 30-40 spheres each sample from different independent field and calculate average size of spheres. For the self-renewal or clonal assays, individual breast cancer sphere cells were plated into 96-well cell culture plates and after 1 day, each well was visually checked to verify the presence of a single cell. The clones were grown, and clone formation was monitored on 1-16 days. Clone size was measured using a phase-contrast microscope with Motic Images Plus 2.0 [34].

### In situ proximity ligation assay (PLA)

Protein-protein interaction was visualized using reliable in situ Proximity Ligation Assay (PLA) technique [35, 36]. Briefly, the cells were fixed with 4% paraformaldehyde and permeabilized with 0.2% Triton X-100. After washing, the cells were blocked with blocking solution. A rabbit polyclonal anti-EGFR antibody (1:200) and anti-STAT3 antibody (1:200) were used for the PLA in situ analysis. All steps of the assay were performed using the Duolink® In Situ PLA® Probe (Sigma) according to the manufacturer's protocol. Amplified PLA signals were analyzed using confocal microscopy and the number of dots per cell were counted using ImageJ software.

### Cross-linking assay

Chemical crosslinking was performed as previously described [37] with 1mM 3,3′-dithiobis (sulphosuccinimidyl propionate) (DTSSP; Thermo-scientific). Briefly, the cells were rinsed with PBS (137 mM NaCl, 0.67 mM KCl, 8 mM Na_2_HPO4, 1.4 mM KH_2_PO4) three times and incubated for 30 min at 4°C with DTSSP in PBS, followed by washing two times with Tris-buffered saline (TBS) (20 mM Tris-HCl, 100 mM NaCl, pH 7.5) prior to use in the studies.

### *In vivo* studies

Mammary tumors were formed by subcutaneous injection of 4T1 cancer cells (1 × 10^6^ cells) into fourth mammary fat pad of athymic Balb/c female nude mice (6-10 weeks old, Orient, Korea). Once tumors were formed, mice were randomly distributed into two groups (n=5/group). Tumors was treated by PHS (1.2 mg of PHS/kg of body) or control vehicle DMSO (equal volume) with i.p injection. Mice who have tumor size more than 100mm^3^ were included in each group. Tumor sizes were measured with a caliper (calculated volume = shortest diameter^2^ × longest diameter/2) at desired intervals. Lung metastasis was analyzed in each case by counting the number of surface metastatic foci in the lung. For tail vein injection, GFP-labeled metastatic MDA-MB231-LM2 cells (1 × 10^6^) were injected into athymic BALB/c nude mice (n=5/group; Orient) by intravenous injection. This study was reviewed and approved by the Institutional Animal Care and Use Committee of Center for Laboratory Animal Sciences, Medical Research Coordinating Center, HYU industry–University Cooperation Foundation. No blinding experiment was done during the animal studies.

### Immunohistochemistry

Mice were sacrificed and tumor tissues were fixed in formalin for the preparation of paraffin sections. Paraffin-embedded tissue sections were deparaffinized in xylene, 95, 90, and 70% ethanol, followed by phosphate buffered saline (PBS). Epitopes were unmasked with 20 mg/mL proteinase K in PBS with 0.1% Triton X-100. Sections were stained with hematoxylin and eosin (H & E). After counter-staining with hematoxylin and clearing with graded ethanol series and xylene, the sections were mounted with Canada balsam. Observation and photography were conducted using an IX71 microscope (Olympus) equipped with the DP71 digital imaging system (Olympus).

### Real time PCR

Cell total RNA was extracted by trizol reagent (Ambion). All qRT-PCR were performed using the KAPA SYBR FAST reagent from KAPA Biosystems (Wilmington, MA, USA) according to the manufacturer's instructions. PCR reactions were carried out using Rotor Gene Q (Qiagen, Korea) and data were expressed as fold change calculated by the ΔΔCt method relative to the control sample. β-actin was used for normalization as a control. All primers used in this study were designed and purchased from Macrogen.

### Molecular modeling

In order to determine the binding affinity of EGFR and PHS, an in-silico substrate-docking experiment was performed using AutoDock Vina [38]. PHS was geometrically optimized using a semi-empirical quantum mechanical method (AM1) and electronic Ligand Builder and Optimization Workbench (eLBOW) [39] prior to modeling docking. Optimized PHS was docked into the Protein Data Bank (PDB) deposited crystallographic structure of the extracellular domain of EGFR (4UV7) or the kinase domain of EGFR (4ZAU). Stochastic global optimization scoring function was used to search for the binding site of EGFR and PHS.

### Statistical analysis

All data are expressed as the means ± Standard deviation (SD) of triplicates. Student's *t*-test was used for comparisons between groups based on an assumption of normal distribution. Levels of significance are indicated as *p < 0.05; **p < 0.01; and ***p < 0.001. All experiments were performed as three independent set of experiments.

## CONCLUSIONS

**Figure 6:**
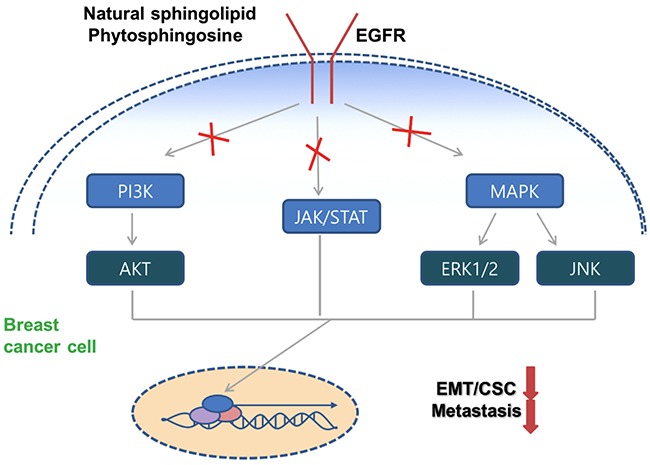
Schematic model of PHS against breast tumor malignancy PHS decreases the EGFR phosphorylation and subsequently deactivates the corresponding downstream signaling to decrease breast tumor progression.

Here, we treated breast cancer cells with a natural sphingolipid PHS, in cell culture conditions and *in vivo* to identify PHS as a novel key driver of the elimination of metastatic cells. Major primary hallmarks of basal-type breast cancers are excessive infiltration and high-grade metastasis [40]. The results presented here indicate that a treatment of breast cancer cells with PHS at micromolar concentrations can exert sustained changes in protein expression levels which lead to EMT without inducing direct cytotoxic effects such as cell death or growth retardation. In tumorigenesis, EMT can enhance the motility and invasiveness of tumor cells, and malignant transformation may be linked to the signaling pathways which stimulate EMT [37]. Lee et al. recently disclosed that an EGFR-targeted therapy can be used to enhance the sensitivity of triple-negative breast cancer cells to chemotherapy [41]. Dysregulation of EGFR pathways by overexpression or constitutive activation can enhance certain aspects of tumor progression, including metastasis, and is associated with poor prognosis in various human malignancies [42, 43]. Interestingly, the present study confirms that PHS may bind to EGFR and suppress its activity in PHS-treated breast cells. According to our molecular modeling results, PHS forms hydrogen bonds with Ser286, Phe287, and Gly288 of the EGFR extracellular domain, whereas in the EGFR domain it forms hydrogen bonds only with Thr790 (Figure 5). In addition, a PHS treatment reduces the interaction of EGFR with the downstream JAK1/STAT3 signaling network in breast cancer cells. We suggest here that this reduction in JAK1/STAT3 activity occurs likely through the direct suppression of EGFR phosphorylation with PHS, with the subsequent regulation of its deactivation (Figure 6). In addition, to mimic basal type phenotype, we also stimulated SKBR3 cells with EGF treatment (EMT inducer). Interestingly, PHS treatment decreases EGFR signaling in these cells also. Several reports have documented a link between EMT and the emergence of a CSC phenotype in human cancers. In breast cancer patients, disseminated breast cancer cells from pleural effusions, which most likely have undergone EMT, are enriched for CD44^high^/ CD24^low^ CSC-like populations [28]. In this regard, we also demonstrated that PHS effectively decreased the CD44^high^ populations and the self–renewal capacity of PHS-treated mammospheres. Moreover, a significant decrease in the CD44 population was noted in basal-type breast cancers (Figure 3). Similarly, exposure to PHS decreased OCT4 protein levels, which have been found to be highly expressed in CD44^high^ populations in breast cancer [44]. In addition to the results of *in vitro* assays, we conducted *in vivo* experiments to provide more direct evidence which supports the findings presented here. Of note, we found that the PHS treatment suppressed lung metastasis in xenograft mice models. Moreover, in a recent report, Wang et al. identified PHS as a biomarker for oral squamous cell carcinoma [45].

Overall, we propose for the first time the use of PHS as a novel therapeutic agent that targets the anti-metastatic abilities associated with EMT and CSCs within basal-type breast tumors, which may be effective at preventing instances of tumor relapse. Notably, we showed that PHS specifically diminishes EGFR activity levels and thus blocks interactions with its downstream JAK1/STAT3 signaling pathways via immunoprecipitation assays. These results invite speculation regarding the role of PHS in the form of an anti-metastatic drug therapy for breast cancer. We also proved that similar to PHS, the EGFR inhibitor AG1478 reduced EMT and CSC markers with an altered EGFR downstream signaling axis (Supplementary Figure 4B). The data presented here support the novel concept of using PHS in the selective targeting of EGFR signaling for breast cancer cells which highly overexpress EGFR. Thus, further study may elucidate the promise of the clinical relevance of PHS for breast cancer patients.

## SUPPLEMENTARY MATERIALS FIGURES



## Abbreviations

PHS: phytosphingosine
CSC: cancer stem-like cells
EMT: epithelial-mesenchymal transition
EGFR: epidermal growth factor receptor

## References

[1] Wykosky J, Fenton T, Furnari F, Cavenee WK (2011). Therapeutic targeting of epidermal growth factor receptor in human cancer: successes and limitations. Chin J Cancer.

[2] Burness ML, Grushko TA, Olopade OI (2010). Epidermal growth factor receptor in triple negative and basal-like breast cancer: promising clinical target or only a marker?. Cancer J.

[3] Rakha EA, El-Sayed ME, Green AR, Lee AH, Robertson JF, Ellis IO (2007). Prognostic markers in triple-negative breast cancer. Cancer.

[4] Guérin M, Gabillot M, Mathieu MC, Travagli JP, Spielmann M, Andrieu N, Riou G (1989). Structure and expression of c-erbB-2 and EGF receptor genes in inflammatory and non-inflammatory breast cancer: prognostic significance. Int J Cancer.

[5] Sainsbury JR, Nicholson S, Angus B, Farndon JR, Malcolm AJ, Harris AL (1988). Epidermal growth factor receptor status of histological sub-types of breast cancer. Br J Cancer.

[6] Radisky DC (2005). Epithelial-mesenchymal transition. J Cell Sci.

[7] Kalluri R, Neilson EG (2003). Epithelial-mesenchymal transition and its implications for fibrosis. J Clin Invest.

[8] Thiery JP, Acloque H, Huang RY, Nieto MA (2009). Epithelial-mesenchymal transitions in development and disease. Cell.

[9] Thiery JP (2002). Epithelial-mesenchymal transitions in tumour progression. Nat Rev Cancer.

[10] Mani SA, Guo W, Liao MJ, Eaton EN, Ayyanan A, Zhou AY, Brooks M, Reinhard F, Zhang CC, Shipitsin M, Campbell LL, Polyak K, Brisken C (2008). The epithelial-mesenchymal transition generates cells with properties of stem cells. Cell.

[11] Maceyka M, Payne SG, Milstien S, Spiegel S (2002). Sphingosine kinase, sphingosine-1-phosphate, and apoptosis. Biochim Biophys Acta.

[12] Peña LA, Fuks Z, Kolesnick R (1997). Stress-induced apoptosis and the sphingomyelin pathway. Biochem Pharmacol.

[13] Cuvillier O (2002). Sphingosine in apoptosis signaling. Biochim Biophys Acta.

[14] Cuvillier O, Pirianov G, Kleuser B, Vanek PG, Coso OA, Gutkind S, Spiegel S (1996). Suppression of ceramide-mediated programmed cell death by sphingosine-1-phosphate. Nature.

[15] Lee JS, Min DS, Park C, Park CS, Cho NJ (2001). Phytosphingosine and C2-phytoceramide induce cell death and inhibit carbachol-stimulated phospholipase D activation in Chinese hamster ovary cells expressing the Caenorhabditis elegans muscarinic acetylcholine receptor. FEBS Lett.

[16] Park MT, Kang YH, Park IC, Kim CH, Lee YS, Chung HY, Lee SJ (2007). Combination treatment with arsenic trioxide and phytosphingosine enhances apoptotic cell death in arsenic trioxide-resistant cancer cells. Mol Cancer Ther.

[17] Park MT, Kim MJ, Kang YH, Choi SY, Lee JH, Choi JA, Kang CM, Cho CK, Kang S, Bae S, Lee YS, Chung HY, Lee SJ (2005). Phytosphingosine in combination with ionizing radiation enhances apoptotic cell death in radiation-resistant cancer cells through ROS-dependent and -independent AIF release. Blood.

[18] Boyer B, Valles AM, Edme N (2000). Induction and regulation of epithelial-mesenchymal transitions. Biochem Pharmacol.

[19] Al-Hajj M, Wicha MS, Benito-Hernandez A, Morrison SJ, Clarke MF (2003). Prospective identification of tumorigenic breast cancer cells. Proc Natl Acad Sci USA.

[20] Sheridan C, Kishimoto H, Fuchs RK, Mehrotra S, Bhat-Nakshatri P, Turner CH, Goulet R, Badve S, Nakshatri H (2006). CD44+/CD24- breast cancer cells exhibit enhanced invasive properties: an early step necessary for metastasis. Breast Cancer Res.

[21] de Beça FF, Caetano P, Gerhard R, Alvarenga CA, Gomes M, Paredes J, Schmitt F (2013). Cancer stem cells markers CD44, CD24 and ALDH1 in breast cancer special histological types. Clin Pathol.

[22] Wright MH, Calcagno AM, Salcido CD, Carlson MD, Ambudkar SV, Varticovski L (2008). Brca1 breast tumors contain distinct CD44+/CD24- and CD133+ cells with cancer stem cell characteristics. Breast Cancer Res.

[23] Becher OJ, Holland EC (2014). Sox2, a marker for stem-like tumor cells in skin squamous cell carcinoma and hedgehog subgroup Q27 medulloblastoma. EMBO.

[24] Koo BS, Lee SH, Kim JM, Huang S, Kim SH, Rho YS, Bae WJ, Kang HJ, Kim YS, Moon JH, Lim YC (2015). Oct4 is a critical regulator of stemness in head and neck squamous carcinoma cells. Oncogene.

[25] Wang Z, Wang N, Li W, Liu P, Chen Q, Situ H, Zhong S, Guo L, Lin Y, Shen J, Chen J (2014). Caveolin-1 mediates chemoresistance in breast cancer stem cells via beta- catenin/ABCG2 signaling pathway. Carcinogenesis.

[26] Ricardo S, Vieira AF, Gerhard R, Leitão D, Pinto R, Cameselle-Teijeiro JF, Milanezi F, Schmitt F, Paredes J (2011). Breast cancer stem cell markers CD44, CD24 and ALDH1: expression distribution within intrinsic molecular subtype. J Clin Pathol.

[27] Giatromanolaki A, Sivridis E, Fiska A, Koukourakis MI (2011). The CD44+/CD24- phenotype relates to ‘triple-negative’ state and unfavorable prognosis in breast cancer patients. Med Oncol.

[28] Shostak K, Chariot A (2015). EGFR and NF-kB: partners in cancer. Trends Mol Med.

[29] Söderberg O, Gullberg M, Jarvius M, KJ Ridderstråle K Leuchowius, Jarvius J, Wester K, Hydbring P, Bahram F, Larsson LG, Landegren U (2006). Direct observation of individual endogenous protein complexes in situ by proximity ligation. Nat Methods.

[30] Yamazaki T, Yoshimatsu Y, Morishita Y, Miyazono K, Watabe T (2009). COUP-TFII regulates the functions of Prox1 in lymphatic endothelial cells through direct interaction. Genes Cells.

[31] Kim RK, Uddin N, Hyun JW, Kim C, Suh Y, Lee SJ (2015). Novel anticancer activity of phloroglucinol against breast cancer stem-like cells. Toxicol Appl Pharmacol.

[32] Hsu SC, Hung MC (2007). Characterization of a novel tripartite nuclear localization sequence in the EGFR family. J Biol Chem.

[33] Kaushik NK, Kaushik N, Yoo KC, Uddin N, Kim JS, Lee SJ, Choi EH (2016). Low doses of PEG-coated gold nanoparticles sensitize solid tumors to cold plasma by blocking the PI3K/AKT-driven signaling axis to suppress cellular transformation by inhibiting growth and EMT. Biomaterials.

[34] Lu F, Wong CS (2005). A clonogenic survival assay of neural stem cells in rat spinal cord after exposure to ionizing radiation. Radiat Res.

[35] Kim JS, Kim EJ, Oh JS, Park IC, Hwang SG (2013). CIP2A modulates cell-cycle progression in human cancer cells by regulating the stability and activity of Plk1. Cancer Res.

[36] Piskunov A, Rochette-Egly C (2012). A retinoic acid receptor RARα pool present in membrane lipid rafts forms complexes with G protein αQ to activate p38MAPK. Oncogene.

[37] Iwamoto R, Higashiyama S, Mitamura T, Taniguchi N, Klagsbrun M, Mekada E (1994). Heparin-binding EGF-like growth factor, which acts as the diphtheria toxin receptor, forms a complex with membrane protein DRAP27/CD9, which up-regulates functional receptors and diphtheria toxin sensitivity. EMBO J.

[38] Trott O, Olson AJ (2010). AutoDock Vina: improving the speed and accuracy of docking with a new scoring function, efficient optimization, and multithreading. J Comput Chem.

[39] Moriarty NW, Grosse-Kunstleve RW, Adams PD (2009). Electronic Ligand Builder and Optimization Workbench (eLBOW): a tool for ligand coordinate and restraint generation. Acta Crystallogr D Biol Crystallogr.

[40] Fulford LG, Reis-Filho JS, Ryder K, Jones C, Gillett CE, Hanby A, Easton D, Lakhani SR (2007). Basal-like grade III invasive ductal carcinoma of the breast: Patterns of metastasis and long-term survival. Breast Cancer Res.

[41] Lee MJ, Ye AS, Gardino AK, Heijink AM, Sorger PK, MacBeath G, Yaffe MB (2012). Sequential application of anticancer drugs enhances cell death by rewiring apoptotic signaling networks. Cell.

[42] Salomon DS, Brandt R, Ciardiello F, Normanno N (1995). Epidermal growth factor-related peptides and their receptors in human malignancies. Crit Rev Oncol Hematol.

[43] Lurje G, Lenz HJ (2009). EGFR signaling and drug discovery. Oncology.

[44] Liu CG, Lu Y, Wang BB, Zhang YJ, Zhang RS, Lu Y, Chen B, Xu H, Jin F, Lu P (2011). Clinical implications of stem cell gene Oct-4 expression in breast cancer. Ann Surg.

[45] Wang Q, Gao P, Wang X, Duan Y (2014). The early diagnosis and monitoring of squamous cell carcinoma via saliva metabolomics. Sci Rep.

