# Supplementary material for: Phytosphingosine exhibits an anti-epithelial–mesenchymal transition function by the inhibition of EGFR signaling in human breast cancer cells
Source: Oncotarget. 2017 Sep 8;8(44):77794–808. doi: 10.18632/oncotarget.20783 (PMC5649924; doi:10.18632/oncotarget.20783)
Supplement: Supplementary file 1 [file oncotarget-08-77794-s001.pdf]

Phytosphingosine exhibits an anti-epithelial–mesenchymal transition function by the inhibition of EGFR signaling in human breast cancer cells

SUPPLEMENTARY MATERIALS

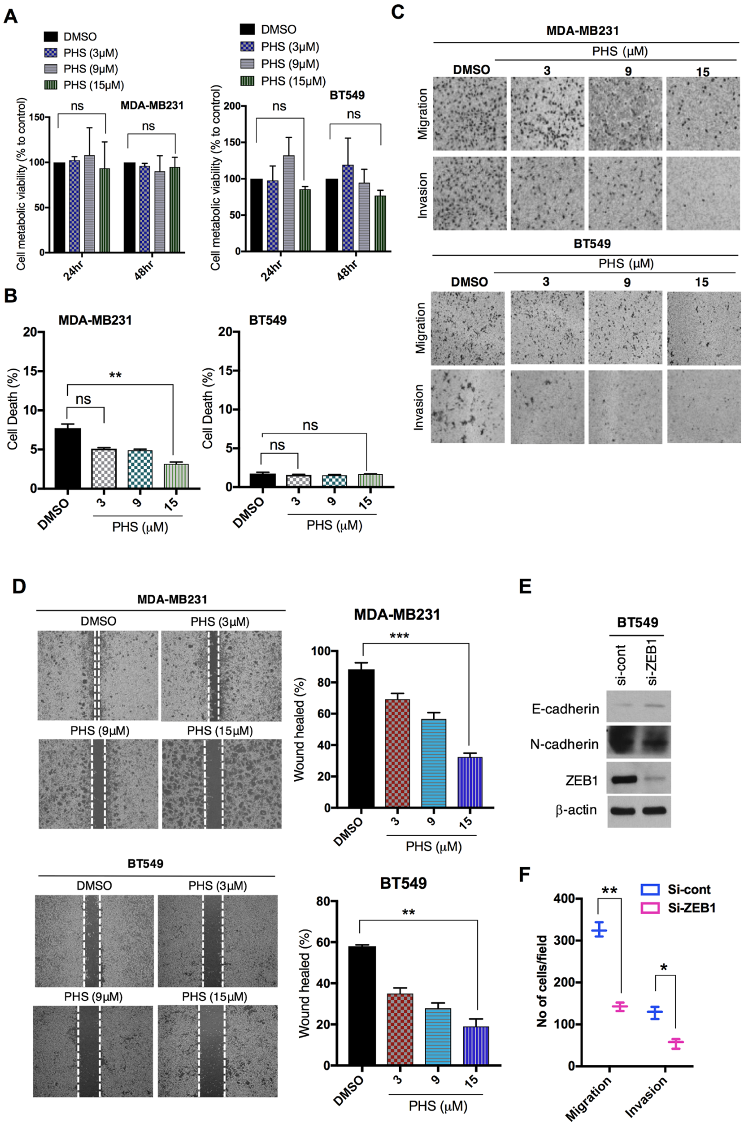

Supplementary Figure 1: PHS critically downregulates EMT of basal type breast cancer cells through ZEB1. **(A)** MTT assays in MDA-MB231 and BT549 cells with increasing concentrations of PHS with incubation time- dependent manner. **(B)** Cell death PI (propidium iodide) analysis in MDA-MB231 and BT549 cells with increasing concentrations of PHS at 48 hr. **(C)** Migration and invasion assays in transwells after DMSO or PHS treatment in MDA-MB231 and BT549 cells at various concentrations. **(D)** Representative images and graphical presentation of wound healing assays in PHS-treated MDA-MB231 (Upper panel) and BT549 cells (Lower panel). **(E)** Western blot for EMT markers such as E-cadherin, N-cadherin in PHS-treated BT549 breast cancer cells that are transfected with siRNA targeting ZEB1. **(F)** Migration and invasion assay in BT549 cells that are transfected with siRNA targeting ZEB1. β-actin was used as a loading control. Error bars represent mean ± S.D. of triplicate samples. $*p < 0.05$, $**p < 0.01$, and $***p < 0.001$.

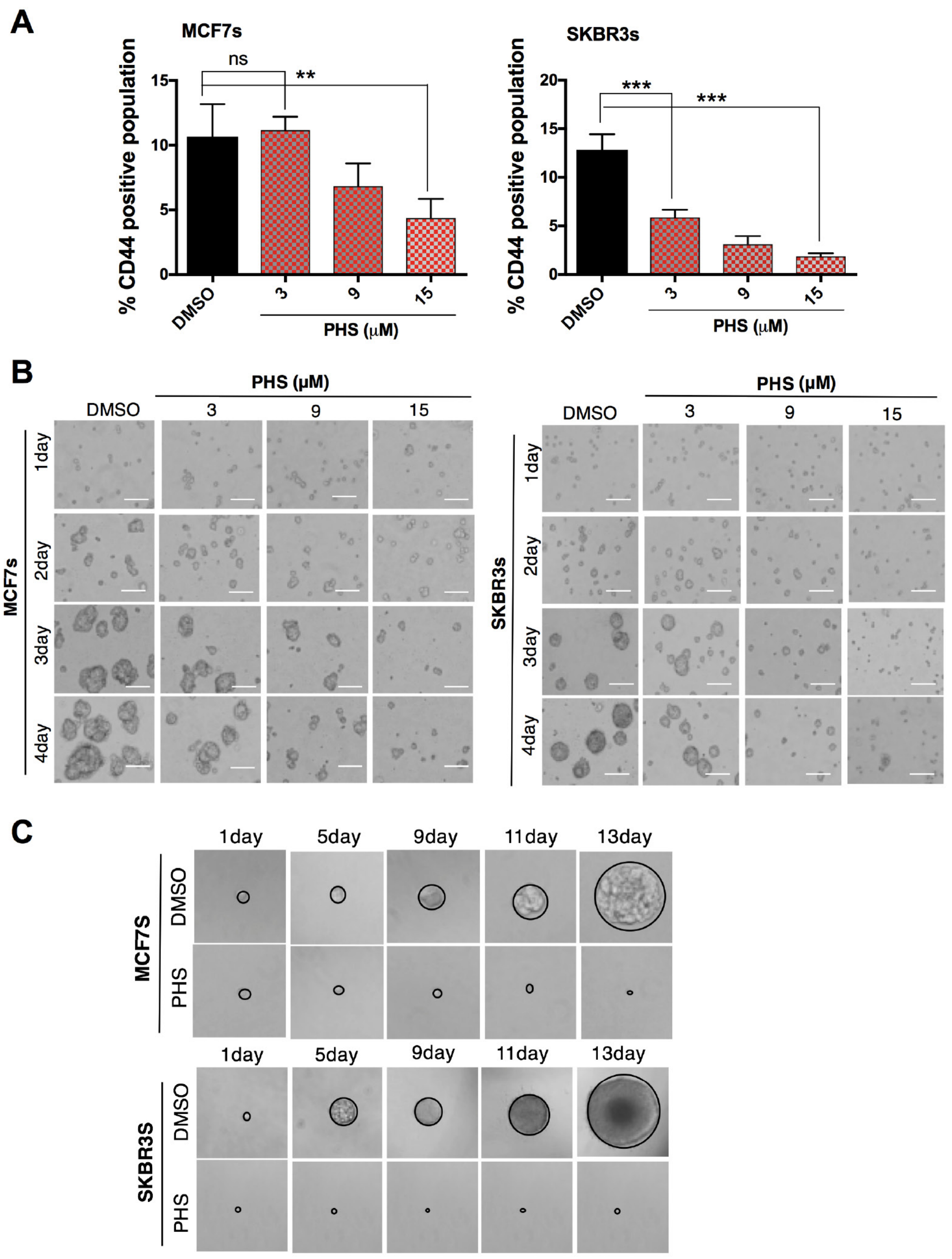

Supplementary Figure 2: PHS decreases CD44 expression and self-renewal capacity of breast cancer cells through targeting OCT4. **(A)** FACS analysis of CD44-PE expression in MCF7 and SKBR3 mammospheres treated with increasing concentrations of PHS. **(B)** Sphere forming assay of MCF7 (Left panel) and SKBR3 (Right panel) sphere cells at 1-4 day cultured with or without PHS (10μM). Scale bar represents 50μm. **(C)** Clonal assays of MCF7 and SKBR3 sphere cells at 1-13 day cultured with or without PHS (10μM).

(continued)

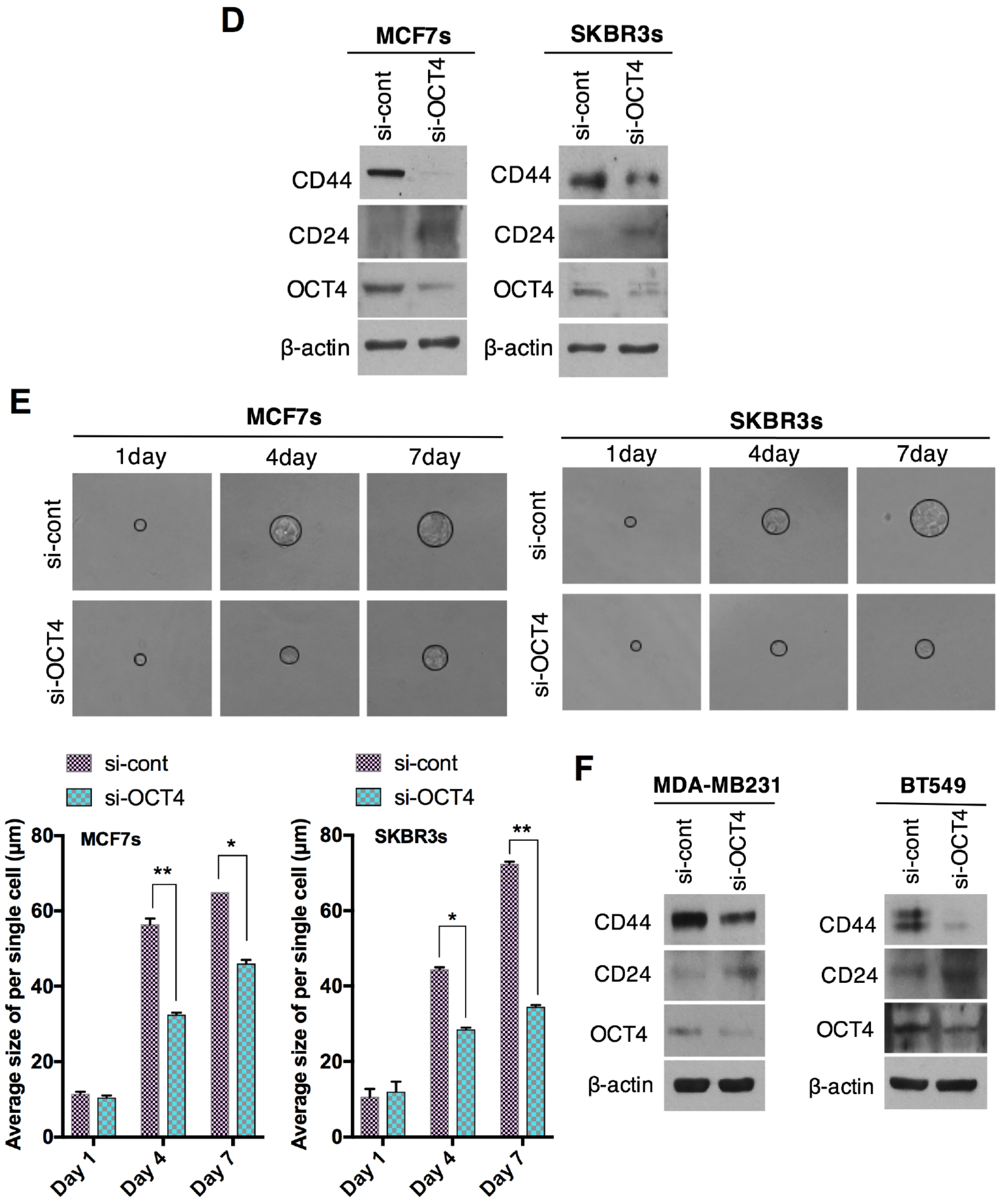

Supplementary Figure 2: PHS decreases CD44 expression and self-renewal capacity of breast cancer cells through targeting OCT4. **(D)** Western blot analysis of CD44 and CD24 protein levels in MCF7 and SKBR3 sphere cultured cell lines after OCT4 silencing. **(E)** Clonal assays of MCF7 and SKBR3 sphere cells at different time points cultured after targeting with si-OCT4. **(F)** Western blot analysis of CD44 and CD24 protein levels in MDA-MB231 and BT549 cell lines after OCT4 silencing. Error bars represent mean ± S.D. of triplicate samples. * $p < 0.05$, ** $p < 0.01$ and *** $p < 0.001$.

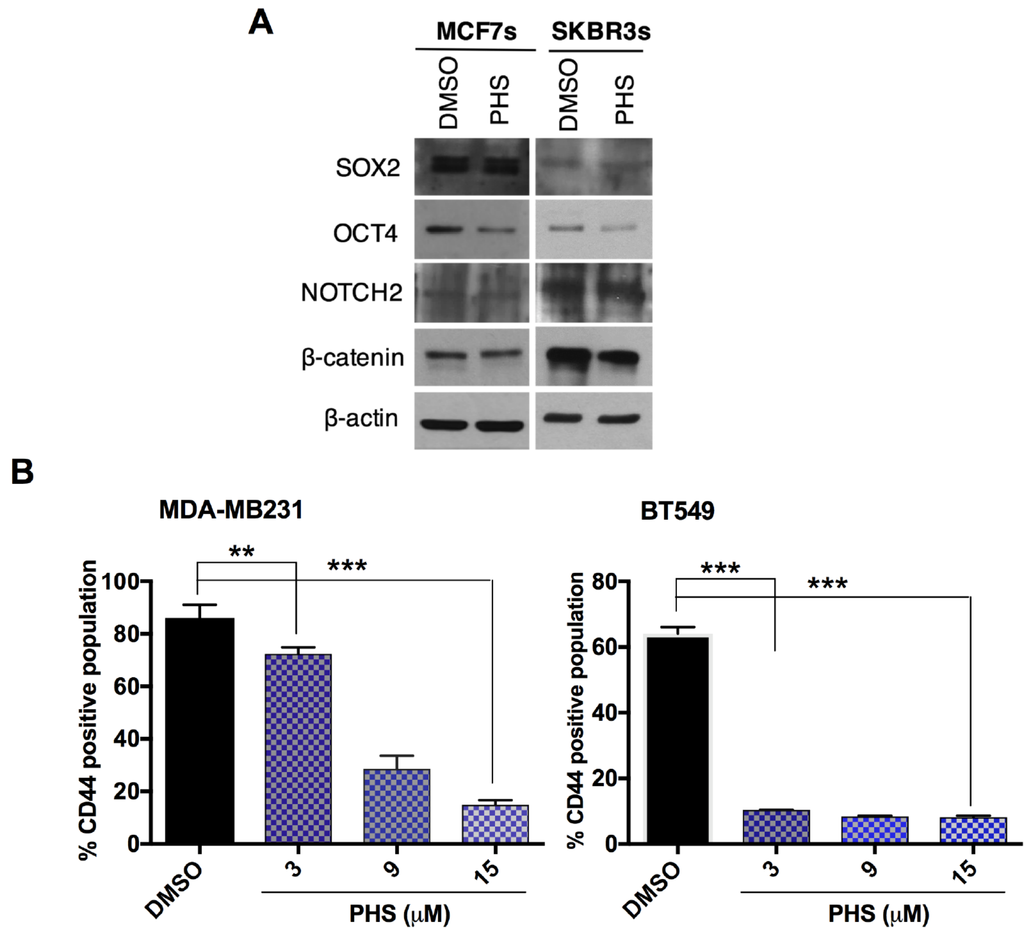

Supplementary Figure 3: PHS reduces the CD44 population in concentration-dependent manner. **(A)** Western blot analysis results for SOX2, OCT4, NOTCH2 and β-catenin in MCF7 and SKBR3 spheres cultured with or without PHS (10μM). **(B)** PHS attenuated the CD44 positive population in basal type breast cancer cells. Error bars represent mean ± S.D. of triplicate samples. ** $p <$ 0.01 and *** $p < 0.001$.

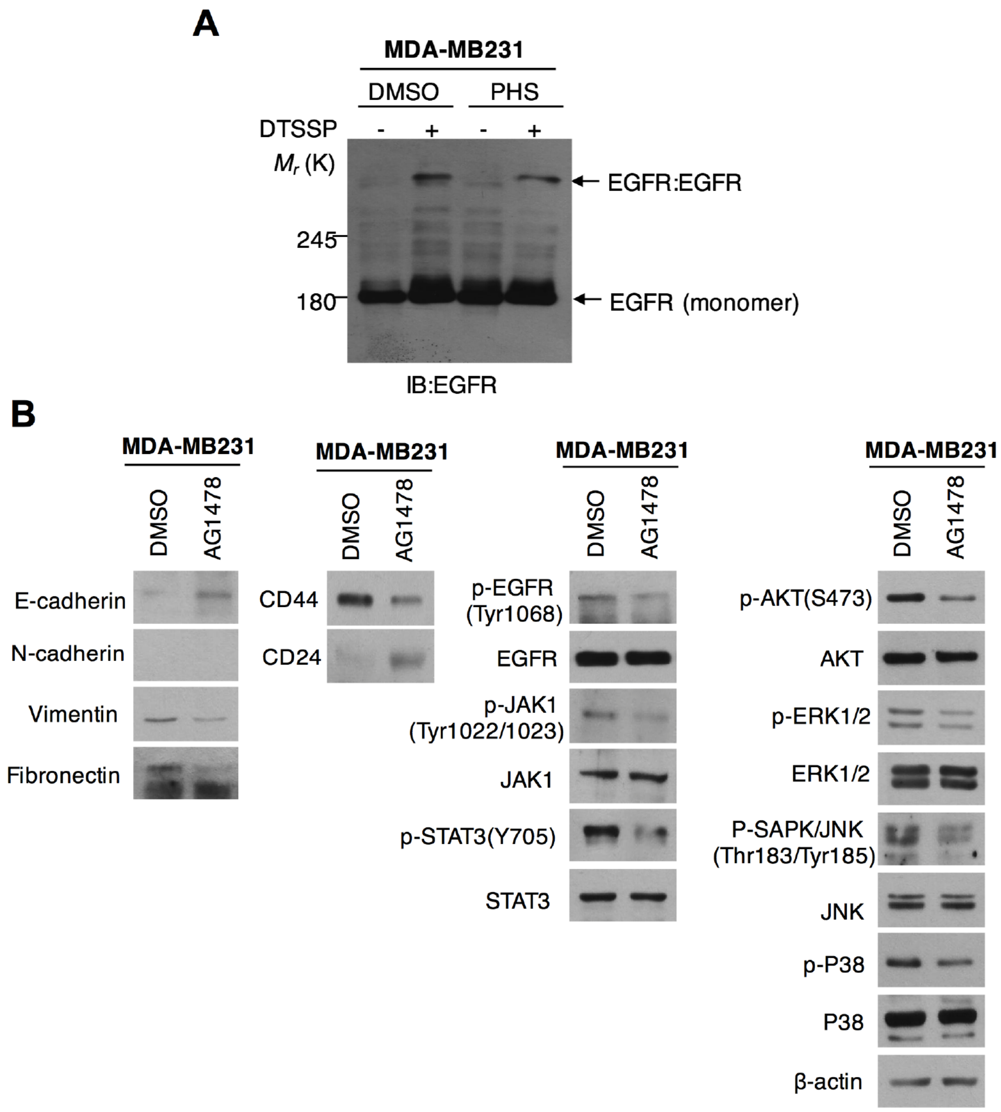

Supplementary Figure 4: PHS does not affect EGFR homodimerization to block EGFR downstream pathway. **(A)** Immunoblot detection using the EGFR antibodies in MDA-MB231 cells treated with PHS followed by DTSSP chemical crosslinking (1mM) for 30 min. **(B)** Western blot analysis in MDAMB-231 for EMT, CSC markers and EGFR downstream signaling axis after EGFR inhibitor (AG1478,10µM) treatment.

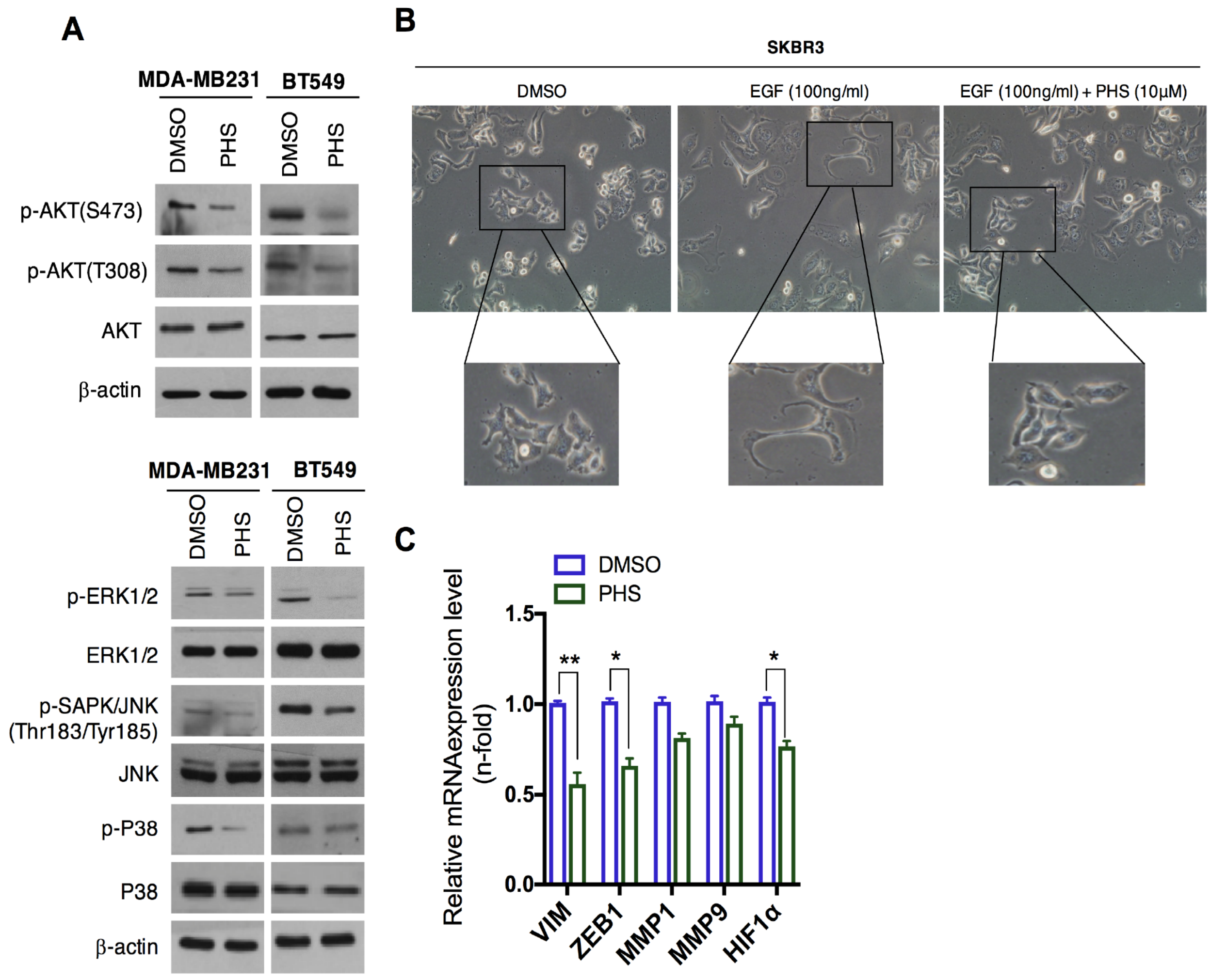

Supplementary Figure 5: PHS decreases MAPK and AKT pathways involved in EMT and decreased invasiveness in breast cancer cells. **(A)** Western blot for MAPK and AKT phosphorylation in MDA-MB231 and BT549 cells after DMSO and PHS treatment (10µM). **(B)** Representative images of EGF and PHS treated SKBR3 cells observed at 20X magnification. Depiction graph is shown to represent the extent of migrated or invaded cells/field. **(C)** qPCR analysis of STAT3 directly targeted genes in DMSO and PHS treated MDA-MB231 cells. Error bars represent mean ± S.D. of triplicate samples, * $p < 0.05$, ** $p < 0.01$, and *** $p < 0.001$.
